# Synthesis and characterization of analcime (ANA) zeolite using a kaolinitic rock

**DOI:** 10.1038/s41598-021-92862-0

**Published:** 2021-06-28

**Authors:** Daniela Novembre, Domingo Gimeno

**Affiliations:** 1 Dipartimento di Ingegneria e Geologia, Università Degli Studi “G.D’Annunzio”, Via dei Vestini 30, 66013 Chieti, Italy; 2 Dept. Mineralogia, Petrologia I Geologia Aplicada, Universitat de Barcelona, 08028 Barcelona, Spain

**Keywords:** Mineralogy, Sustainability, Environmental chemistry

## Abstract

Analcime is nowadays an important component in dental porcelain systems, in heterogeneous catalysis, in the nanoelectronic field, in selective adsorption and in stomatology (dental filling and prosthesis). Analcime synthesis from an impure, silica-rich kaolinite rock coming from Romana (Sassari, Italy) is here presented. A synthesis protocol is proposed that aims to make an improvement of synthesis conditions compared to the past. The hydrothermal treatment is in fact here achieved without aging times and without the use of sodium silicate or other additional silica source reported in the literature. Lower calcination temperature, synthesis temperature and crystallization time are verified in this work. The kaolin is subjected to calcination at the temperature of 650 °C and then mixed with NaOH. The experiment is performed at ambient pressure and 170 ± 0.1 °C. The degree of purity of analcime is calculated in 97.57% at 10 h. Analcime is characterized by X-ray diffraction, infrared spectroscopy, scanning electron microscopy, inductively coupled plasma optical emission spectrometry and thermal analysis. Density is also calculated. Cell parameters and the amount of amorphous phase in the synthesis powders is estimated with quantitative phase analysis using the combined Rietveld and reference intensity ratio methods. The experimental conditions make the synthesis protocol particularly attractive from an economic point of view. Also this work does not use a commercial kaolin but silica-rich impure kaolinitic rock from a disused quarry. This further reduces the costs of the experimental protocol. It also gives the protocol an added value, as the synthesis of a useful mineral is obtained through the valorization of an otherwise unused georesource. Both chemical and physical characterization of analcime is satisfactory making the experimental protocol very promising for an industrial transfer.

## Introduction

The structure of zeolites consists of three-dimensional frameworks of SiO_4_ and AlO_4_ tetrahedra arranged to form channels containing water and exchangeable cations (sodium, potassium, calcium etc.). Analcime (ANA) zeolite is the smallest-pore zeolite, and it exhibits a compact structure compared to other zeolites with an idealized unit cell of Na_16_Al_16_Si_32_O_96_·16H_2_O^1^; its complex structure, build of corner sharing [SiO_4_] and [AlO_4_] tetrahedron, creates irregular channels and some cavities occupied by the exchangeable Na-ions in the crystal lattice^2^. Analcime is usually of cubic singony, space group *Ia3d*^3–6^. Saha^6^ described the cubic unit cell composed of four-, six-, and eight-membered oxygen rings forming three non-intersecting channels. Every sodium ion is surrounded by four oxygens ions and two water molecules, which make up a distorted octahedron^7^. Yokomori and Idaka^8^ report that every pseudocubic or average cubic analcime is really trigonal (R-3). Orthorombic natural analcime is also reported^9^. Analcime occurs in various geological environments. Type H analcime is formed under hydrothermal conditions by dissolution of source materials such as nepheline, plagioclase, albite, quartz, volcanic glass and recrystallization from hydrothermal solutions. A particular case of Analcime which is formed under hydrothermal conditions is that which is formed by cationic exchange K–Na from Leucite^10^; this analcime is called X type Analcime. Morphologically it retains the leucite habit, but it is dusty and often the crystal core is still leucite^11^. This cation exchange reaction requires 3 days at 300 °C and 1 Kbar and the use of Leucite crystals of at least 100 microns size, as well as an activation energy of 8 kcal/mol^12^. The thermal^11^ and morphological^13^ analyses allow to distinguish the type of analcime (H or X). Then there is the I type analcime which segregates itself directly from the magma and occurs inside igneous rocks^14^.

Analcime exhibits a variety of applications in technology, especially in selective adsorption in waste water treatments^15–18^ and heterogeneous catalysis^19–21^; in particular, the incorporation of transition elements (Fe, Co, Ni, Cu, Ti and V) in the zeolite framework, or in the porous system as compensating cations, allows to create diverse catalytic sites^22,23^. Analcime is also successfully used as fertilizer dispenser in agriculture^15^, in the nanoelectronic field^24^, in stomatology, i.e. in ceramics for denture^25,26^.

Even if Analcime is found in nature, abundant supplies of this mineral occur in limited regions of the world. For this reason, recent research is moving towards the synthesis of this mineral by using different sources of silica and alumina.

Usually, analcime is synthesized under hydrothermal conditions using aluminosilicate clear solutions or gels, in the presence of an alkaline medium and at temperature variable from 100 to 310 °C. Some studies demonstrates that the more Na_2_O is in the initial raw materials, the lower the temperature at which analcime crystallizes^27^. The alternative route is to synthesize analcime from cheap local materials, i.e. by the conversion of natural glasses^28^, conformed ashes^29^, rhyolitic tuff^30^, rice husk^31–35^, rice husk and perlite^36^, clinker^37^, quartz syenite powders^38^, k-felspar^39^ and clays^22,40,41^ which represent highly reactive raw materials. However not only analcime prevails among the reached synthesis^42^.

As regards the use of clays, kaolin is the material already tested in the synthesis of analcime. Among clay minerals, in fact, kaolinite is the most used phyllosilicate in the zeolitic synthesis because of its particularly large supply and availability and the well-known activity of thermally treated kaolin clays (metakaolin) when treated with alkali-based reagents^43–49^. Rios et al.^50^ treated kaolinite with NaOH and obtained co-crystallization of analcime and other zeolites; Hegazy et al.^22^ synthesized analcime by hydrothermal reaction of kaolin and commercial sodium silicate solution at 200 °C for 24 h under autogenous pressure; Atta et al.^40^ synthesized analcime from kaolin after 72 h aging and 24 h reaction time at temperature of 180 °C; Jamil et al.^41^ mixed Kaolinite, Ludox 40 and NaOH and get analcime after 30 min at 160 °C in a microwave system; Moraes et al.^51^ treated calcined kaolin and diatomaceous earth with NaOH at 210 °C for 24 h and obtained analcime with minor amounts or residual quartz; Kwaky-Awuah et al.^52^ synthesized powders of LTA, analcime and X zeolite starting from Kaolin and bauxite; Ramirez-Zamora et al.^33^ synthesized polimineralic powders of analcime by fly ash and kaolinite; Abdul-Moneim et al.^53^ treated kaolin with NaOH at 170 °C for 36 h and obtained co-crystallizations of analcime, cancrinite and sodalite.

As is evident from the results of the previous attempts aimed at the synthesis of the analcime starting from kaolin, one of the fundamental problems is not being able to synthesize monomineral powders of analcime; the mineral is, in fact, often present as an accessory in the synthesis of other zeolites, or in coexistence with phases associated to the starting material^51^. This fact (not to obtain monomineralic products) is the same that prevents extensive use of natural analcime. The coexistence in the synthesis powders of analcime with other mineralogical phases has made its characterization difficult up to now, limiting this only to morphological and X-ray diffractometric observations. In few cases^22,40,41^ authors declare to obtain monomineralic powders of analcime, but it must also be said that none of them has actually conducted a study aimed at characterizing the purity of the synthetic product, i.e. at investigating the possible presence of unreacted and/or amorphous material. The lack of these data prevents an industrial transfer of the synthesis protocols; industry requires at least 90% pure synthesis powders.

Given the situation, the purpose of this work is to synthesize analcime by testing a natural rock, i.e. a kaolinitic rock coming from Romana (Italy). This kaolin has already been successfully used in the past in the synthesis of useful minerals^47–49^. Romana kaolin has the advantage of being characterized by an excess of silica compared to common commercial kaolin, being its mineralogical composition made of kaolinite plus minor amounts of quartz and opal-crystobalite^47^. This peculiar characteristic gives the material a Si/Al ratio greater than 1 and it makes it suitable for the synthesis of minerals characterized by and Si/Al ratio higher than 1:1, like analcime. Because of this, the use of Romana kaolin avoids the addition of sodium silicate or other silica sources, and it makes the synthesis protocol simpler and above all cheaper than in the past.

In particular, this work aims to improve the previous attempts made in the past for the synthesis of the analcime from kaolin by avoiding co-crystallization of mineral phases, aging times and by lowering: (1) synthesis temperature (2) crystallization time and (3) kaolin calcination temperature. Systematic samplings during the experimental run enable to follow the progress in the crystallization of the mineral phases and allows to determine the time at which the climax in the crystallization is reached. The degree of purity of the synthesized powders expressed in terms of absence of amorphous phase and/or impurities coming from the natural kaolinite sample is here defined through a quantitative phase analysis approach using the combined Rietveld and reference intensity ratio methods.

## Materials and methods

The kaolin used in this study comes from a mine loca ted in Romana (Sassari, Italy). For the chemical composition of kaolin and its mineralogical, morphological and spectroscopic characterization, see Novembre et al.^47^. The kaolin was triturated, and the sandy fraction was separated by retention in a sieve; then the fraction below 90 μm was collected, suspended in distilled water, sonicated, and centrifuged for separation of the silt fraction and collection of the clay fraction^49^. Preliminary calcination of kaolin was carried out in open porcelain crucibles heated in a Gefran Model 1200 furnace (Gefran Spa, Brescia, Italy) to the calcination temperature (650 °C) at a pressure of 1 atm. The heating rate of the sample was 1.5 °C s^−1^. Once the calcination temperature was reached, the crucibles were left in the furnace for 2 h and then removed and cooled at room temperature. The NaOH used in the synthesis protocol was purchased from Riedel-de Haën (Honeywell Riedel-de Haën, Bucharest, Romania). The purity of the reagent was of 99%. As explained above, kaolin of Romana is characterized by an excess of silica compared to the common stoichiometry of commercial kaolin^47^, and do not require addition of sodium-silicate. 2 g of metakaolinite have been directly dissolved in 20 ml of a NaOH (8%) solution. The initial mixture had the composition: 6.25 SiO_2_–1.00 Al_2_O_3_–3.6 Na_2_O. The mixture was homogenized for two hours with a magnetic stirrer. Then it was put inside a stainless-steel hydrothermal reactor and heated at 10 °C/min until the desired temperature (170 °C). Synthesis products were sampled periodically from the reactor, filtered with distilled water and dried in an oven at 40 °C for a day.

Kaolin and products of synthesis were analyzed by powder X-ray diffraction (XRPD); the instrument was a Siemens D5000 operating with a Bragg–Brentano geometry (CuKα = 1.518 Å, 40 kV, 40 mA, 4°–120° 2theta scanning interval, step size 0.020° 2theta). Identification of analcime and relative peak assignment was performed with reference to the following JCPDS code: 00-019-1180. Both the crystalline and amorphous phases in the synthesis powders were estimated using quantitative phase analysis (QPA) applying the combined Rietveld and reference intensity ratio (RIR) methods; corundum NIST 676a was added to each sample, amounting to 10% (according to the strategy proposed by Novembre et al.^47,49^ and the powder mixtures were homogenized by hand-grinding in an agate mortar. Data for the QPA refinement were collected in the angular range 5°–110° 2theta with steps of 0.02° and 10 s step^−1^, a divergence slit of 0.5° and a receiving slit of 0.1 mm.

Data were processed with the GSAS software^54^ and the graphical interface^55^ starting with the structural model proposed by Gatta et al.^56^ for analcime. The following parameters were refined: background parameters, zero shift, cell parameters and peak profiles.

Morphological analyses were obtained by means of scanning electron microscopy (JEOL JSM-840 served by a LINK Microanalysis EDS system, with operating conditions of 15 kV and window conditions ranging from18 to 22 mm)^57^.

Induced coupled plasma optical emission spectroscopy technique (ICP-OES, Perkin Elmer Optima 3200 RL) was performed on synthesized powders through previous fusion (Pt meltpot) in lithium meta-tetra borate pearls and subsequent acid solubilisation and analytical determination^58^.

Density of Analcime was calculated by He-picnometry using an AccuPyc 1330 pycnometer^59^.

The infrared analysis was performed with a spectrometer FTLA2000, served by a separator of KBr and a DTGS detector; the source of IR radiation was a SiC (Globar) filament. Samples were treated according to the method of Novembre et al.^60^ using powder pressed pellets (KBr/sample ratio of 1/100, pressure undergone prior determination 15t/cm^2^); spectra were processed with the program GRAMS-Al (GRAMS/AI ™ Spectroscopy Software, Thermo Scientific Company).

Differential thermal analysis (DTA) and thermogravimetry (TG) were performed using a Mettler TGA/SDTA851e instrument (10°/min, 30-1100 °C, sample mass of ~ 10 mg, Al_2_O_3_ crucible) (Mettler Toledo, Greifensee, Switzerland).

## Results

**Figure 1:**
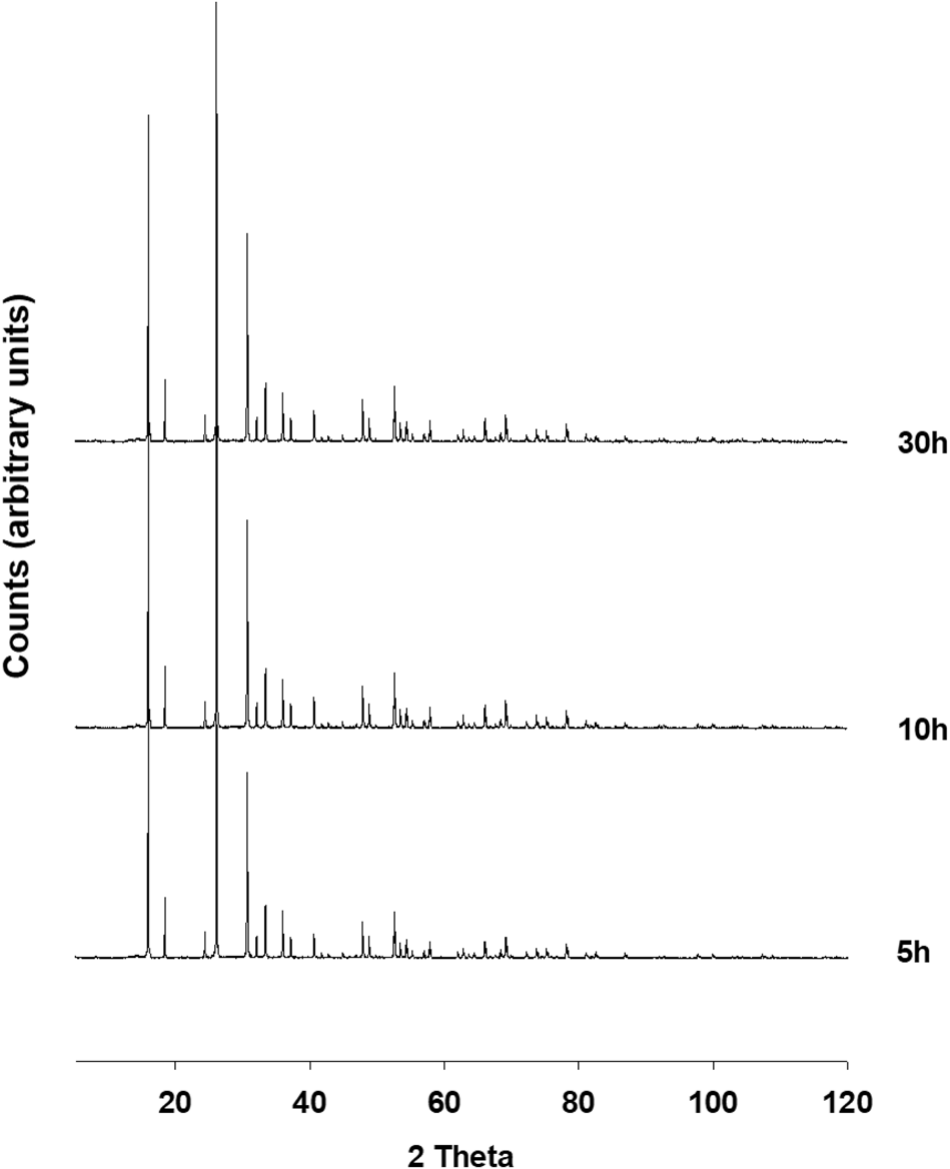
X-ray diffractometric sequence of the synthesis run at 170 °C.

Results of XRPD analyses performed on the synthesis run conducted at 170° are illustrated in Fig. 1.

Synthesis of analcime is evident at 5 h. The existence field of the ANA zeolite is very large, in fact the phase remains isolated for a long time; peaks grow in height until reaching the maximum intensity at 10 h. The intensity of the peaks remains unchanged even at 30 h, proving that the climax of crystallization is reached only 10 h after the start of the thermal treatment. Results of the QPA analyses conducted on samples at 5, 10 and 30 h are illustrated in Table 1. Analcime percentage increases over time at the expense of the amorphous component and reaches its climax at 10 h (97.57%). There is no substantial change in the percentages of the crystalline fraction versus the amorphous one passing from 10 to 30 h. It must be said that the nature of the silica source used in the synthesis and the impurities present in it are reported to have a significant effect on the purity of the final products^61,62^. This is certainly the major limitation found in mineral synthesis procedures starting from georesources. In the particular case of analcime synthesis, Navickas et al.^42^ report that the amount of analcime in the synthesis products depends on the nature of the raw materials and Atta et al.^40^ report metallic impurities coming from the rice husk and kaolinite used as starting material in the synthesis protocol. The QPA analysis allows to exclude the presence of accessory mineralogical phases and / or impurities coming from the starting kaolinite.

**Table 1:** Results of the QPA analyses conducted on samples synthesized at 170 °C.

Sample + 10% corundum Nist 676a	5 h	10 h	30 h
R_wp_	0.19	0.18	0.18
R_p_	0.14	0.15	0.14
CHI^2^	2.08	2.18	2.17
Space group ANA	*Ia-3d*	*Ia-3d*	*Ia-3d*
*a* (Å)	14.2071 (0.0051)	14.2041 (0.0037)	14.2052 (0.0035)
*b* (Å)	10.0515 (0.0005)	10.0536 (0.0018)	10.0539 (0.0021)
*c* (Å)	10.0418 (0.0007)	10.0427 (0.0004)	10.0416 (0.0006)
% amorphous	6.53 (13)	2.43 (11)	2.47 (11)
ANA	93.47 (17)	97.57 (14)	97.53 (14)

**Figure 2:**
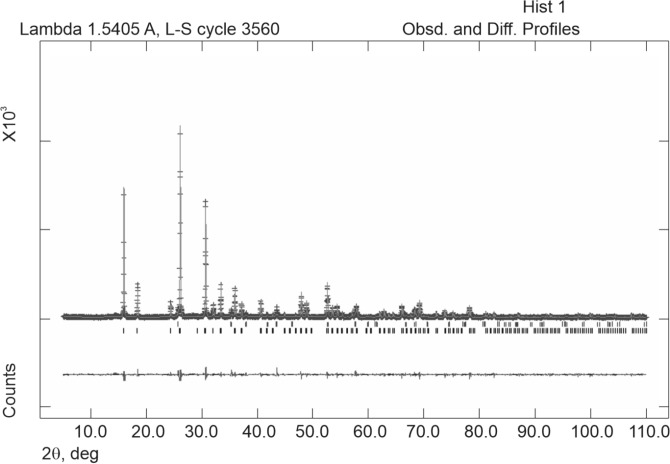
Rietveld refinement plot: Observed ( +) and calculated profiles and difference plot for analcime zeolite (10 h at 170 °C) and corundum NIST 676a with tick marks at the position of the Bragg peaks. From the bottom: analcime zeolite, corundum NIST 676a.

For the sample at 10 h the observed and calculated profiles and difference plots for analcime and corundum NIST 676a are reported in Fig. 2. Cell parameters of analcime, refined with cubic simmetry space group *Ia-3d*, remain constant within error as a function of the experimental run time. The results of the Rietveld refinements provide cell values that are in good agreement with the structural model proposed by Gatta et al.^56^.

**Figure 3:**
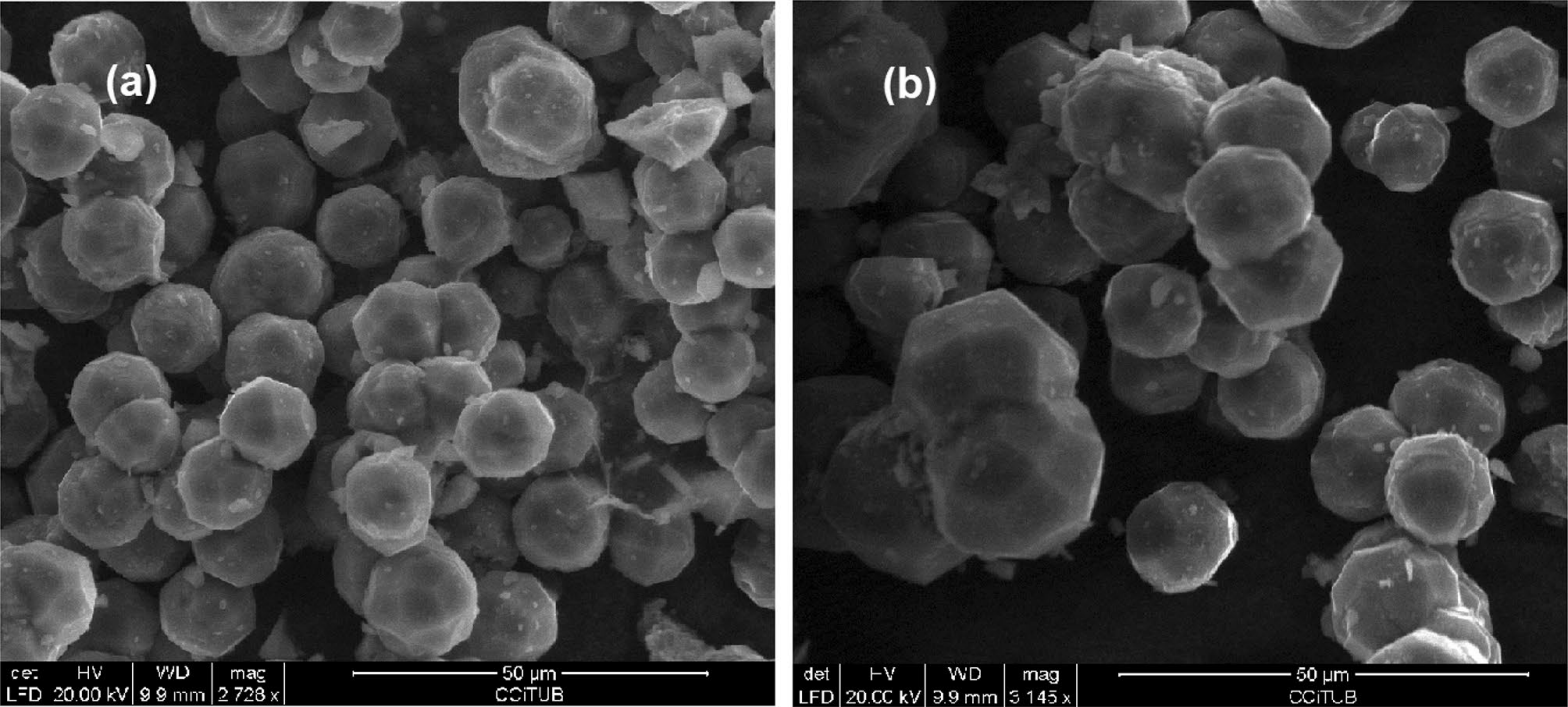
SEM images of analcime zeolite crystals obtained at 5 h (**a**) and at 10 h (**b**) of the synthesis run.

Figure 3a and b reports SEM images of analcime crystals at 5 h and 10 h, respectively. It results an average maximum length of crystals observed to be around 25 µm. This result is very satisfying when compared with the size of the crystals obtained by other authors in the past. As an example, Jamil et al.^41^, synthesize analcime from kaolinite reaching crystal sizes of 5–7 µm, while Hegazy et al.^22^ testify average dimensions of 10 µm. Chemical analysis performed on samples at 10 h (170 °C) resulted in the stoichiometry of Na_6.00_Al_5.98_Si_12.02_O_36_. The density of analcime from the sample at 10 h (170 °C) was determined to be 2.261(5) g/cm^3^.

**Figure 4:**
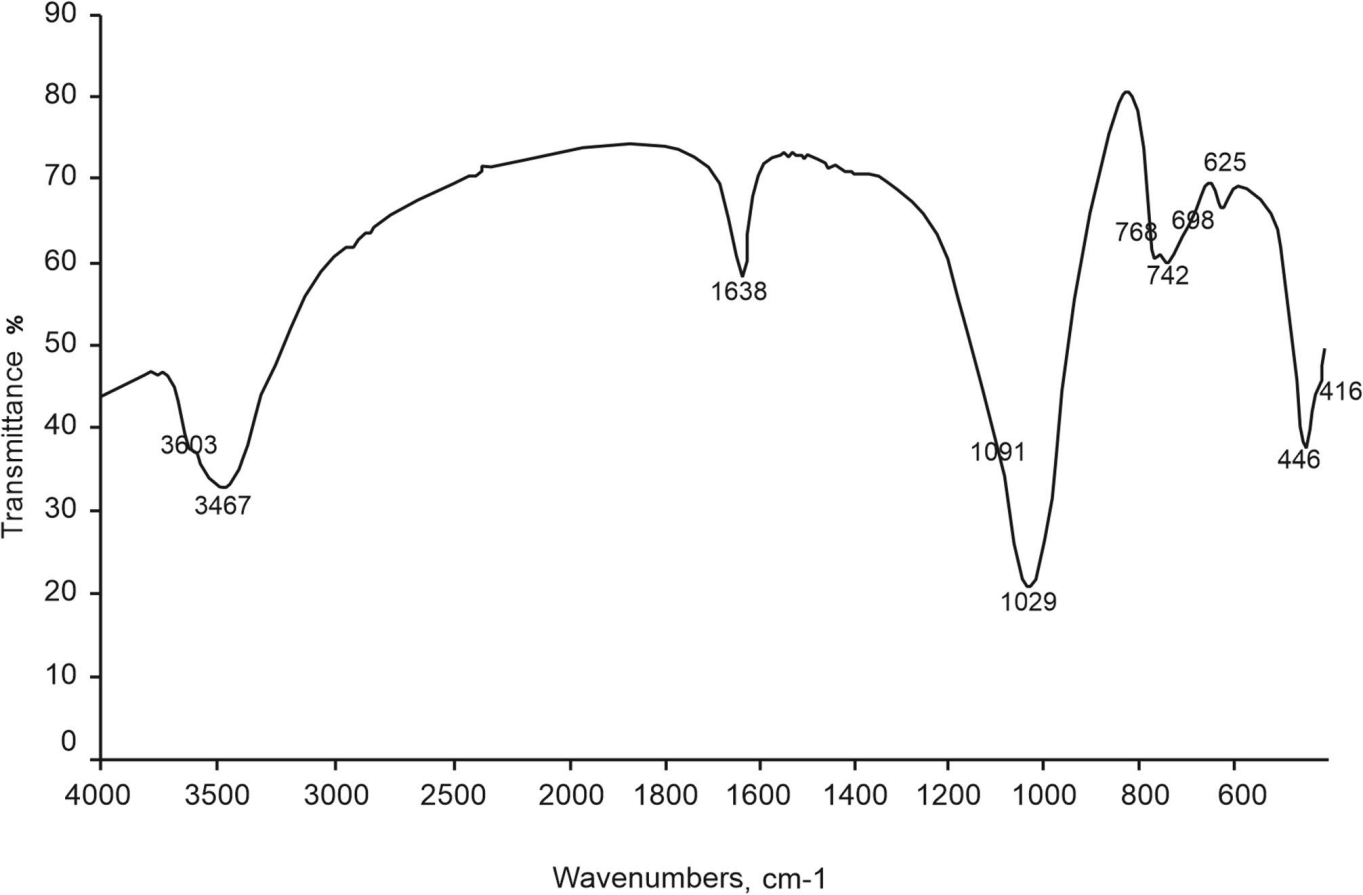
IR spectrum of the analcime at 10 h (170 °C).

Further characterizations were carried out on the sample at 10 h (170 °C). Figure 4 illustrates the infrared spectrum of the sample. Information on the structure of the mineral can be inferred from the vibrational frequencies of the lattice observed in the range between 4000 and 400 cm^−1^. Data reported in Fig. 4 are coherent with those available in the literature^24,40,63^. The significant broad peaks are located at 3467 and 1638 cm^−1^ for O–H stretching and bending, respectively. In particular, the band at 3467 cm^−1^ is associated to the asymmetric stretching mode of water coordinated to the edges of the channels, while the band at 1638 cm^−1^ is assigned to the zeolitic water in the channels of zeolite^64^. The bands at 1029 and 1091 cm^−1^ are attributed to the stretching mode of O–Na–O. The bands at 768, 742, 698 and 625 cm^−1^ are attributed to Si–O–Si symmetric stretching vibration, i.e. to the symmetric stretching vibration of 4-membered rings. Abdul-Monheim et al.^53^ noticed that these bands occur at relatively high wave numbers in the pseudo lattice band range as these rings contain the lowest number of all rings occurring in the zeolite structure. Bands at 446 and 416 cm^−1^are characteristic of of O–Si–O bonding mode, i.e. to the typical bending vibrations of 4-membered rings.

**Figure 5:**
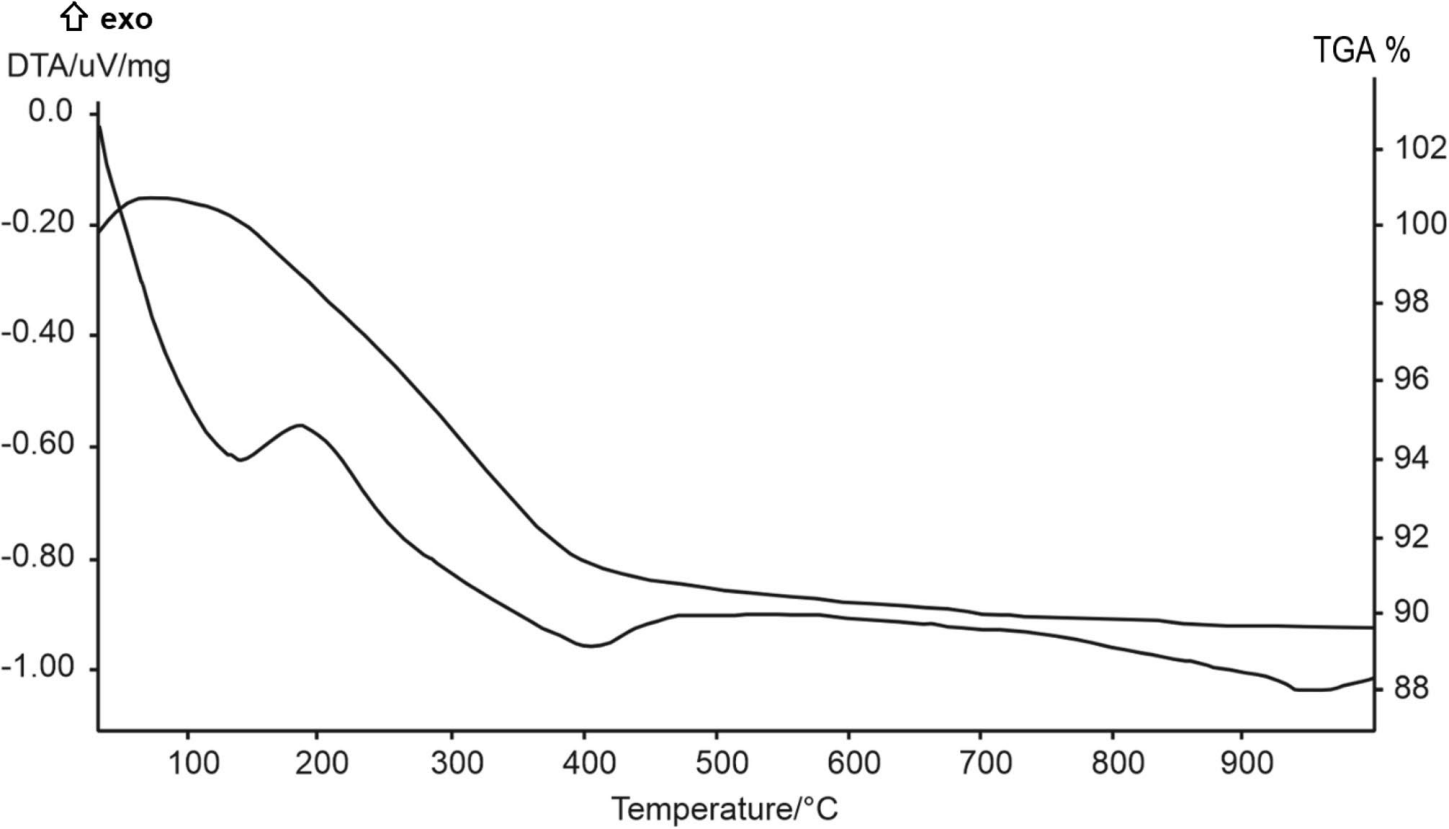
DTA-TG analysis of the sample at 10 h (170 °C).

Thermogravimetric analysis conducted on samples at 10 h (170 °C) revealed a gradual and continuous water loss up to 1000 °C (Fig. 5). In particular three well defined dehydration steps are observed at 140, 415 and 980 °C. This three step-dehydration process here observed is in agreement with findings by Sandoval et al.^37^. The position and number of peaks can be attributed to different compensating cation-water binding energies and also to the different energy related to the diffusion of desorbed water^53^. Moreover, the amount of desorbed water is related with the number of compensation cations in the framework of the zeolite^65^. In particular, the first two observed peaks in Fig. 5 correspond to removal of physical absorbed and occluded water. The loss at 980 °C is due to gradual removal of water in micropores. A total weight loss of 13.5% is obtained, in agreement with data of Sandoval et al.^37^ and Hegazy et al.^22^.

## Conclusion

A goal of fundamental importance that research today seeks to pursue is the green synthesis of minerals achieved by using naturally derived reagents. At the same time, research seeks to reduce synthesis time and temperature to create inexpensive protocols from an economic point of view. This work describes the synthesis of analcime using a kaolinitic rock coming from Romana (Sassari, Italy). Analcime has already been synthesized in the past from kaolin^22,33,40,41,51–53,65^. One of the fundamental problems identified in the attempts to synthesize analcime starting from kaolinite is the enormous difficulty of obtaining the mineral isolated from other phases. Only in a few cases some authors report having synthesized monomineral powders of analcime^22,40,41^. When our results are compared with the past literature, a reduction of calcination temperature of kaolinite, of the synthesis temperature, and of crystallization time is evident. Furthermore, no aging time is required in the synthesis protocol. In addition, our protocol does not require for the use of sodium silicate or other silica sources^41^. Romana kaolin is characterized by an excess of silica compared to pure kaolin^47^; this makes the experimental procedure cheaper and faster.

Hegazy et al.^22^ operate a calcination temperature of kaolinite of 900 °C, while we reduced it to 650 °C.

Hegazy a et al.^22^ got analcime at 24 h at 200 °C, and Atta et al.^40^ got Analcime after 72 h aging and 24 h reaction time at 180 °C; in our work ANA zeolite crystallizes at only 5 h at 170 °C. In our case the existence field of the zeolite is very large, in fact no phase replaces it in the time interval 5–30 h. The dimensions of the crystals obtained are larger when compared with previous authors^22,40^ and also greater than those obtained by the microwave synthesis method^41^, in spite that this is a more performing method that considerably reduces the time and crystallization temperatures (30 min at 160 °C).

In mineralogical synthesis processes in general, but when synthesizing starting from natural reagents in particular, great attention must be paid to characterizing the degree of purity of the synthetic products. In fact, amorphous residues or even impurities of the starting material can often be present in the final ones, or even results in the desired phase is not isolated, but in coexistence with others. Industry requires at least 90% purity. It must be said that as far as we know none of the authors in the past has estimated the degree of purity of the synthetic powders. In our opinion, from this it is not possible to establish whether these are representative of the climax of crystallization, or to exclude the presence of minor other phases and/or amorphous residues.

The degree of success of an experiment is here established from calculation of the percentage of crystallization *vs.* amorphous material and other impurities. In our case, already at 5 h of the synthesis run, 93.47% is reached, which is already a sufficient achievement for the parameters required by industry. Then at 10 h it exceeds 97%. This percentage remains unchanged for a long time, therefore the stability range of the mineral is wider than that obtained by the previous authors in the literature. All other characterization, i.e. chemical-physical, morphological, and spectroscopic characterization of experimental products testifies the efficacy of the experimental protocol proposed here. All these results suggest that transfer to an industrial production scale would be easily possible.

Last but not least, this work does not use a commercial kaolin but a kaolinite rock from a disused quarry. This further reduces the costs of the experimental protocol. It also gives the protocol an added value, as the synthesis of a useful mineral is obtained through the enhancement of an otherwise unused georesource. In addition, as Romana's kaolin is characterized by an excess of silica with respect to commercial kaolin, this study opens the way to the use of so-called "impure" kaolins and demonstrates that are a better natural product (till now rejected in most of cases, and therefore, cheaper) in the synthesis processes of useful minerals characterized by a Si/Al ratio higher that 1:1.
